# The Specific Molecular Composition and Structural Arrangement of *Eleutherodactylus Coqui* Gular Skin Tissue Provide Its High Mechanical Compliance

**DOI:** 10.3390/ijms21165593

**Published:** 2020-08-05

**Authors:** Justin Hui, Shivang Sharma, Sarah Rajani, Anirudha Singh

**Affiliations:** 1 Department of Biomedical Engineering, Johns Hopkins University, Baltimore, MD 21218, USA; jhui6@jhu.edu (J.H.); srajani2@jhu.edu (S.R.); 2 Department of Chemical & Biomolecular Engineering, Johns Hopkins University, Baltimore, MD 21218, USA; ssharm55@jhu.edu; 3 Department of Urology, The James Buchanan Brady Urological Institute, The Johns Hopkins School of Medicine, Baltimore, MD 21287, USA

**Keywords:** collagen, elastin, bladder, compliance, microarchitecture, biomimicry

## Abstract

A male *Eleutherodactylus Coqui* (*EC*, a frog) expands and contracts its gular skin to a great extent during mating calls, displaying its extraordinarily compliant organ. There are striking similarities between frog gular skin and the human bladder as both organs expand and contract significantly. While the high extensibility of the urinary bladder is attributed to the unique helical ultrastructure of collagen type III, the mechanism behind the gular skin of *EC* is unknown. We therefore aim to understand the structure–property relationship of gular skin tissues of *EC*. Our findings demonstrate that the male EC gular tissue can elongate up to 400%, with an ultimate tensile strength (UTS) of 1.7 MPa. Species without vocal sacs, *Xenopus Laevis* (*XL*) and *Xenopus Muelleri* (*XM*), elongate only up to 80% and 350% with UTS~6.3 MPa and ~4.5 MPa, respectively. Transmission electron microscopy (TEM) and histological staining further show that *EC* tissues’ collagen fibers exhibit a layer-by-layer arrangement with an uninterrupted, knot-free, and continuous structure. The collagen bundles alternate between a circular and longitudinal shape, suggesting an out-of-plane zig-zag structure, which likely provides the tissue with greater extensibility. In contrast, control species contain a nearly linear collagen structure interrupted by thicker muscle bundles and mucous glands. Meanwhile, in the rat bladder, the collagen is arranged in a helical structure. The bladder-like high extensibility of *EC* gular skin tissue arises despite it having eight-fold lesser elastin and five times more collagen than the rat bladder. To our knowledge, this is the first study to report the structural and molecular mechanisms behind the high compliance of *EC* gular skin. We believe that these findings can lead us to develop more compliant biomaterials for applications in regenerative medicine.

## 1. Introduction

For millions of years, animals have developed many unique organs for the sole purpose of attracting a suitable mate. These organs take advantage of phenomena such as color [1], scent [2], sound [3], and visual size [4]. Many of these phenomena are exemplified and exploited by many male frogs (*anuran*) to attract a suitable female [5]. Most commonly known is the sound of a frog and the iconic inflation of its gular skin (vocal sac). The characteristic inflation of the gular skin serves as a visual stimulus during mating season and facilitates the energetic mechanical effectiveness of air movement during calls [5]. Investigating the extraordinary inflating action of the gular skin can uncover novel mechanisms behind their ability to stretch and guide the development of artificial compliant biomaterials for regenerative medicine.

Most families in the order *anuran* have inflatable gular skin, such as *Hylidae*, *Eleutherodactylidae*, and *Leptopelis;* however, the size, shape, and color can vary significantly between species [6,7,8]. Commonly associated with frogs is the single inflated gular skin located under the floor of the gular; however, some species can have two external vocal sacs or no external vocal sac at all [5]. The vocal sac is imperative to the success of the male frog in attracting its female counterpart. *Anuran* vocal sacs play the same role in enhancing the calling effectiveness of the male frog to penetrate their often heavily forested habitats. The calling process begins with the frog filling its lungs with air and subsequently passing it over its vocal cords to produce a call. To reduce the time required to inhale after every call, the vocal sac stores and pushes air back into the lungs, effectively removing the need to inhale [5]. The vocal sac is able to increase the energetic mechanical efficiency of this process because of its elastic property. It stores the energy while the muscles push the air into the sac and release the energy to push the air back into the lungs, much like a helical spring or rubber balloon. The evolution of this elastic organ allows frogs to be very efficient in their calling and produce thousands of calls per night [5]. Doing so enables females to pinpoint the male’s location for mating.

In this study, we focused on the male *Eleutherodactylidae Coqui* (*EC*) for its single inflatable gular skin. The highly stretchable nature of the gular skin in frogs is reminiscent of organs in several other species, such as the body of *Lagocephalus Gloveri* [9] and *Diodon Holocanthus* (pufferfish) [10], *Nerodia Sipedon* (snake) gular [11], the gular skin of *Fregata Magnificens* (frigate bird) [12], and the urinary bladder [13]. These organs possess the material phenomena known as elasticity and compliance that allow them to expand to such great volumes. Compliant materials can stretch easily under low forces, as opposed to stiff materials, which require significant force to deform the material slightly. These mechanical features originate from the composition of structural proteins and their architectures.

Elastin is a protein often associated with higher elasticity and compliance in tissues [14,15]. Several groups have incorporated elastin to develop highly elastic biomaterials, with the caveat that most methods include crosslinkers that are inherently cytotoxic (i.e., glutaraldehyde, hexamethylene diisocyanate) [16]. Other strategies used either peptide materials [17,18,19] or micro/nanofiber reinforced materials [20,21] to enhance elasticity and compliance; however, they are still unable to match the high extensibility of the urinary bladder. The inability of these materials to recapitulate the biomechanics of the bladder warrants further investigation into other mechanisms of elasticity and compliance, such as tissue ultrastructure. Using scanning electron microscopy (SEM), Murakumo et al. identified a unique helical architecture of collagen type III within the bladder, suggesting a role of collagen architecture in its biomechanics [13]. Although some groups have incorporated micro/nanofibers [20,21] into the material, the fibers are deposited in its fully elongated state and simply act to increase tensile strength instead of compliance. While it is possible to electrospin intertwined nanofibers [22], the authors of the study did not apply their technique in scaffold construction. We believe that the configuration or microarchitecture of collagen in tissues likely plays a significant role. Thus, the characterization of collagen microarchitectures in several compliant tissues in nature could reveal alternative ultrastructures that may be translated into biomaterial design.

**Figure 1:**
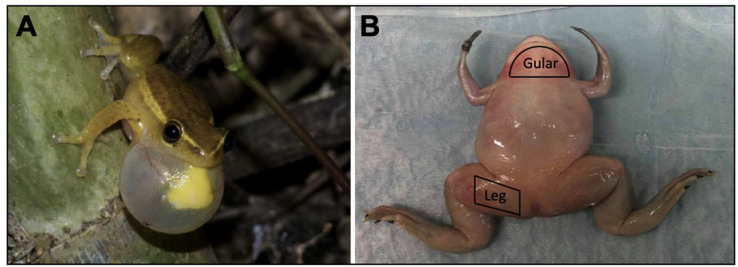
(**A**) A male *Hyperolius Cinnamomeoventris,* with inflated gular skin tissue to resonate its mating call. Reproduced from [5]. (**B**) Gross visual of tissue dissection areas. Shown here is the Xenopus Laevis specimen.

Here, we characterize the commonly observed biomechanics and biocomposition of the gular skin, shown in Figure 1A. To fully understand the mechanism behind the highly inflatable gular skin, we performed experiments to characterize the tensile strength, tissue morphology, ultrastructure, and collagen/elastin content. We further compared the male EC gular skin tissue with frogs without a visually inflatable gular skin, namely *XL*, *XM*, female *EC*, and the male rat urinary bladder. In addition, leg tissues (Figure 1B) were dissected from the frogs and underwent the same analyses to identify the key features that allow the *EC*’s gular skin to be functionally unique. We performed TEM, tensile tests, histology, and biochemical assays. We demonstrate that the gular skin of *EC* can achieve comparable elongation to the rat bladder and is more compliant than *Xenopus Laevis* (*XL*) and *Xenopus Muelleri* (*XM*).

Furthermore, we identified a unique and more sophisticated collagen ultrastructure in *EC* gular skin, different from the helical structure observed in the urinary bladder. *EC* gular skin has a combination of several structures, such as layering, crimping, and twisting. The significance of these features is further emphasized by the statistically insignificant amount of elastin in most of the test samples. We believe that biomimicry of the collagen microstructure present in the *EC*’s gular skin may provide researchers with an alternative solution to reconstruct a more mechanically significant scaffold for regenerative medicine.

## 2. Results

### 2.1. Mechanical Properties

**Figure 2:**
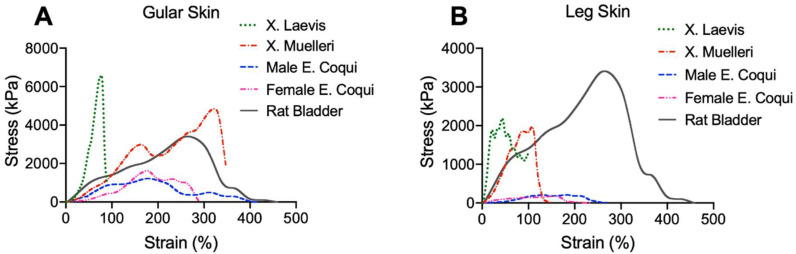
Representative uniaxial stress–strain curves of (**A**) gular skin tissue and (**B**) leg skin of different anuran species and their comparison to the rat bladder.

The uniaxial tensile tests illustrate marked differences between the frogs of genus *Xenopus* and those of *Eleutherodactylidae*, in addition to location-dependent properties. Figure 2 shows representative stress vs. strain and membrane tension characteristics of the species’ gular skin tissue and leg skin tissue, with rat bladder as a comparison. Mean and standard deviation (SD) values are summarized in Table 1. It is evident in both Figure 2A,B that *Xenopus* tissues were stiffer relative to those of *Eleutherodactylidae,* as observed by the steep rise in the stress vs. strain curve before ultimate tensile strength (UTS) was reached. As shown in Table 1, there is a general trend of leg tissue being stiffer at 20% elongation, indicated by a higher secant modulus.

Furthermore, male *EC* tissues had lower secant modulus values than all samples tested, highlighting their uniqueness. Interestingly, gular skin tissue from *XM* displayed similar elongation at failure when compared to male *EC*, which were, on average, 350% and 398%, respectively. However, *XM* required much higher stress to elongate the same amount as *EC*, shown in Figure 2A. *XL* behaved as expected, with a very steep slope (indicative of a high secant modulus) followed by quick failure at 104% and 108% for gular and leg tissue, respectively, in stark contrast to samples from *EC* (gular: 398%, leg: 348%). Gular tissue of the male *EC* exhibited an average ultimate tensile strength (UTS) of 1263 ± 134 kPa, half that of its female counterpart (2142 ± 1789 kPa) and a quarter that of the *XL* (4461 ± 2215 kPa) and *XM* (4156 ± 1973 kPa). Comparisons between male and female *EC* show that male gular tissue exhibited higher elongation at break, of ~400% and ~340%, respectively. Figure 2B illustrates the stress vs. strain behavior of leg tissue, displaying similar trends as gular tissue. *Xenopus* samples had higher secant modulus values than *Eleutherodactylidae* in addition to a lower elongation at failure. The most striking observation is the clear difference between the final elongations between the tissue types. In all cases, the gular tissue of a single specimen had a greater elongation than the leg tissue.

Further comparisons with male rat bladder illustrated that there is a closer biomechanical resemblance of male *EC* gular tissue to the urinary bladder. The rat bladder had an average elongation of ~410%, similar to that of male and female *EC* tissues. Additionally, it had a mean peak stress of ~3000 kPa, highlighting its ability to retain urine and prevent failure.

We determined the secant modulus for assessing the stiffness of the sample. From Table 1, it is evident that leg tissues were generally stiffer than the same species’ gular tissue, indicated by a higher mean value of the secant modulus. Male EC exhibited slightly higher mean secant modulus values of 373 kPa and 318 kPa for gular and leg tissue, respectively. As expected, the male EC had lower secant modulus values compared to the other species, and XL leg tissue exhibited the highest mean secant modulus of 4863 kPa. In general, leg tissues had higher secant modulus values than gular tissues. However, these trends did not show any statistical significance when compared to the rat bladder (Table 1).

### 2.2. Tissue Morphology

**Figure 3:**
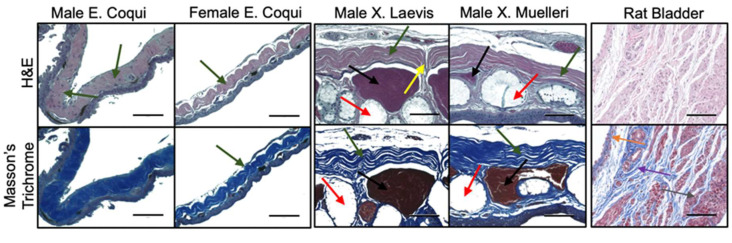
Tissue morphology by histology of gular tissue dissected from various species of frogs. Scale bar 100 µm. Muscle bundles (black arrows), mucous glands (red arrows), collagen structure (green arrows), perpendicularly aligned collagen (yellow arrows), urothelium (orange), lamina propria (purple), and detrusor muscle (grey).

H&E and trichrome-stained gular tissue cross-sections are shown in Figure 3. Gular tissue dissected from frogs belonging to the genus *Xenopus* are substantially thicker and contain a higher density of muscle bundles and mucous glands. Observations of the collagen structure between the families showed marked contrasts. The collagen structure in the male *EC* had a continuous and crimp structure, in stark contrast to the female *EC*, *XL*, and *XM*, which have more spread out crimp structures. Additionally, the tissue sample of the genus *Xenopus* has a nearly linear collagen structure. Interestingly, *XL* and *XM* have distinct regions where the collagen is perpendicularly aligned. These areas seemingly discretize the tissue into collagen segments.

**Figure 4:**
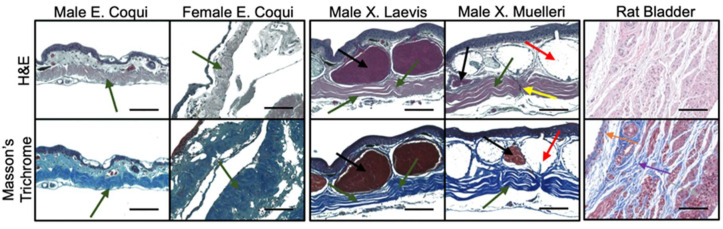
Hematoxylin and eosin (H&E) staining of leg tissue dissected from various species of frogs. Scale bar 100 µm. Muscle bundles (black arrows), mucous glands (red arrows), collagen structure (green arrows), perpendicularly aligned collagen (yellow arrows), urothelium (orange), lamina propria (purple), and detrusor muscle (grey).

Histological cross-sections of leg tissue show similar trends with the collagen structures between the frog species. *EC* tissue has a more pronounced crimp structure compared to *XL* and *XM*. Once again, the muscle bundles and mucous glands constitute a more substantial portion of the tissue in *Xenopus* samples. Collagen in the male *EC* leg has a less compact crimp structure than that found in the gular tissue. Similar to the gular tissue of the *Xenopus* samples, the collagen structure is more linear and has minimal crimping. Leg tissues do not exhibit the same inflatable function as the gular tissue; therefore, it does not require the crimped collagen structure. Lastly, sectioning of fresh rat bladder showed an abundance of muscle bundles with intermittent collagen fibers, dissimilar to the frog tissues. Collagen ultrastructures found in gular tissues were not visually identified in the rat bladder seen in Figure 3 and Figure 4.

### 2.3. Tissue Ultrastructure

**Figure 5:**
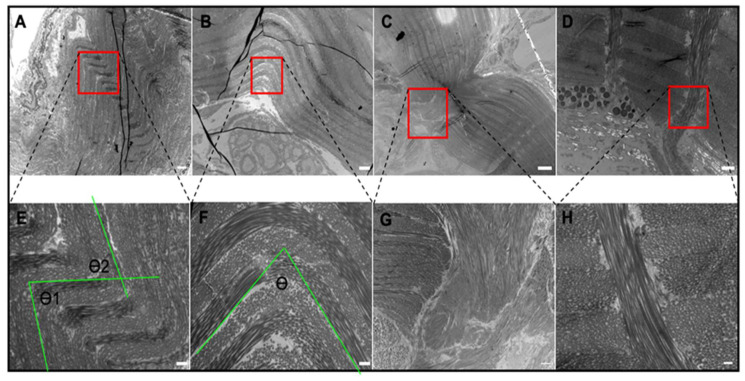
TEM images of gular tissue for (**A**) male EC, 3400×. Scale bar 2 µm; (**B**) female EC, 3400×. Scale bar 2 µm; (**C**) XL, 1000×. Scale bar 10 µm; (**D**) XM, 4200×. Scale bar 10 µm; (**E**) male EC, 13,500×. Scale bar 500 nm. Green lines represent the crimp angle measurements (θ1 = 80°, θ2 = 70°). (**F**) Female EC, 13,500×. Scale bar 500 nm. Green lines represent the crimp angle measurements (θ = 77°). (**G**) XL, 3400×. Scale bar 2 µm. (**H**) XM, 13,500×. Scale bar 500 nm. Red boxes indicate the area magnified.

TEM was used in this study to properly observe the collagen microstructure and orientation, shown in Figure 5. Images at 3400× magnification reinforce the observations from histology and show unique patterns in the *EC* gular tissue compared to *XL* and *XM*. Higher magnification images (13,500×) were obtained from the areas highlighted with the red box in the first row of Figure 5A–D and shown in the second row (Figure 5E–H). At higher magnification, the intricate orientation and design of the collagen layers can be identified. Figure 5E,F show a tri-dimensional collagen arrangement defined by the waviness, transitions from circular to rectangular cross-section within a layer, and contrast differences showing axial twisting. Although the layering pattern is present in the *Xenopus* tissues, other patterns are not observed. In contrast, there are discontinuities within the collagen layers of the *Xenopus* tissues shown in Figure 5C,D.

Furthermore, Figure 5E,F can be used to identify key physical characteristics of the crimped collagen structure. Firstly, there is a clear “layering” structure within the bulk tissue, which indicates an alternating collagen bundle orientation across the tissue. In the male *EC*, a collagen layer (Figure 5E) is approximately 500 nm thick, whereas the female *EC* is 667 nm thick. The two figures also show very clearly that the male *EC* gular tissue has more layers within the same field of view. Secondly, calculations were conducted to identify the crimp angle of the collagen bundles. As shown in Figure 5, the angles between the two folded layers of the male *EC* tissues are 80° and 70°, while there is a single fold of 77° for the female EC tissues. Those of the *Xenopus* genus were virtually 0°, meaning that they were nearly straight throughout the tissue.

**Figure 6:**
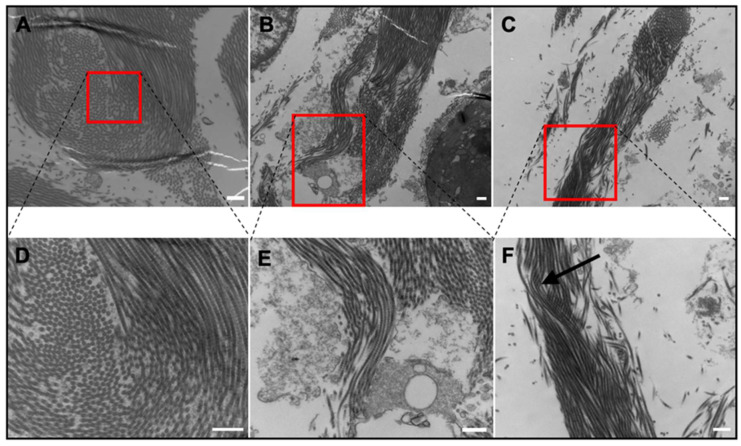
TEM images of the rat bladder. (**A**) Alternating collagen orientation. 17,500×. Scale bar 500 nm. (**B**) Crimp structure. 13,500×. Scale bar 500 nm. (**C**) Helical structure. 9700×. Scale bar 500 nm. (**D**) Magnified alternating collagen orientation. 33,000×. Scale bar 500 nm. (**E**) Magnified crimp structure. 24,500×. Scale bar 500 nm. (**F**) Magnified helical structure. 17,500×. Scale bar 500 nm.

Some similar features were observed in the rat bladder and are shown in Figure 6. Rat bladder and *EC* gular skin had several features in common, such as out-of-plane orientation and crimping (Figure 6A,B). As shown in Figure 6D, collagen strands change from longitudinal to circular cross-sectional orientation, similar to those seen in Figure 5E,F. However, the rat bladder lacks the layered structure found in *EC* tissues. Rat bladder had a thick collagen bundle (2.5 µm), shifting its orientation all at once, whereas *EC* tissues had smaller bundles stacked on top of each other and alternating their orientation. Figure 6B,E resemble a crimped collagen bundle. This was a short bundle and was seldom identified across the tissue. As expected, a helical structure was observed in the rat bladder (Figure 6C,F). The twisted collagen strands identified in Figure 6F show very clearly the formation of spring-like structures, reminiscent of those found in the human bladder. These observations were frequently found throughout the tissue cross-section.

### 2.4. Biochemical Analysis

**Figure 7:**
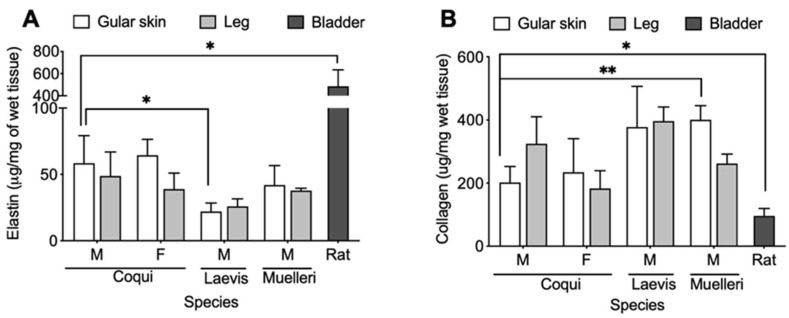
(**A**) Elastin content and (**B**) collagen content for the respective tissue sample. M: male, F: female. *p* < 0.05 (*) and *p* < 0.01 (**).

As shown in Figure 7, *EC* tissues had, on average, much higher elastin concentrations than the *Xenopus* samples. On the other hand, there are minimal differences between the elastin content in male and female *EC* tissues. As summarized in Table 2, there was an average of 58.5 ± 16.9 and 64.5 ± 9.8 μg/mg of wet tissue in male and female gular skin, respectively. The elastin content found in *EC* gular tissues was higher than the *Xenopus* gular tissues, with 22.1 ± 5.2 and 42.1 ± 11.9 μg/mg of wet tissue for *XL* and *XM,* respectively. In general, leg tissues contained less elastin than the gular tissues, and, once again, *EC* samples contained more elastin than *Xenopus* samples (Figure 7A). Collagen assays show the opposite trend, with *EC* tissues containing less collagen than *Xenopus*. Male *EC* gular tissue contained an average of 202.0 ± 41.3 μg/mg of wet tissue and female *EC* had 234.5 ± 86.7 μg/mg of wet tissue. These values were much lower than those of *XL* and *XM*, with 377.8 ± 86.7 μg/mg and 400.8 ± 36.5 μg/mg of wet tissue, respectively (Figure 7B). As expected, male *EC* leg tissue contained more collagen than the gular tissues; however, this trend was not found in the other samples. Although *EC* tissues had more elastin than *Xenopus* tissues, the similar concentrations of elastin between male and female *EC* further imply the importance of the collagen structure in the extensibility of the tissue. Lastly, the average elastin to collagen ratio was calculated to show that the *EC* gular tissue had higher elastin to collagen ratios of 0.29 and 0.27 for males and females, respectively. In stark contrast, *Xenopus* gular tissues were 0.06 and 0.1 for *XL* and *XM,* respectively. As a comparison, the male rat bladder had significantly more elastin and less collagen, with averages of 484.8 ± 121.9 and 96.1 ± 19.4 μg/mg of wet tissue, respectively. Rat bladder has approximately eight times more elastin than the male EC. As expected, the rat bladder had five times more elastin than collagen, unlike frog tissues, where the opposite is observed.

## 3. Discussion

To understand the fundamental mechanisms behind the iconic inflatable gular tissue seen in select frog species, in the present study, we analyzed multiple frog tissues. Among all tested frog tissues, *EC* tissue displayed lower secant moduli in uniaxial tensile tests, more sophisticated collagen microarchitectures, and higher elastin to collagen ratios. These mechanical characteristics allow the tissue to meet the functional requirements of the gular tissue to move air between the vocal sac and the lungs mechanically. The low UTS (1263 ± 134 kPa) of the male *EC* gular tissue reflects the easily extensible tissue, allowing the frog to inflate its gular skin without excessive force. Interestingly, the gular tissue in all species had a higher UTS when compared to its leg tissue. This is likely indicative of the need for the tissue to withstand repeated stress from fast air movement. Furthermore, gular tissue from male *EC* had the highest average elongation of nearly 400% compared to the other species. In contrast to expectations, the gular tissue of *XM* was able to elongate, on average, 350% ± 9% of its original length, surpassing the female *EC* but not achieving the same elongation of the male *EC*. On the other hand, *XL* elongated substantially less, with only an average of 104% ± 17%. The leg tissues of all species had lower peak stress and strain values compared to their gular tissue counterparts. In all species, leg skin tissue displayed half the peak stress of the gular skin except for male *EC*. The male *EC* gular skin had a UTS of 1263 kPa while the leg skin had 193 kPa. This trend shows that the gular skin is a mechanically more durable material than that of the leg. These UTS values also occurred at smaller strain values than those of the gular skin. Male *EC* gular skins’ UTS occurred at 187%, compared to its leg tissue at 83%, as shown in Table 1. The high elongation and moderate UTS of *EC* gular tissue reflect its role as a mechanically dynamic tissue. Its frequent expansion and loading require it to sustain higher stress while still elongating, much like the rat urinary bladder. Gular tissue of the male *EC* and *XM* had comparable elongations to the male rat bladder (~412%). The rat bladder also had UTS averaging ~3000 kPa, higher than tissue from *EC* but lower than *XM* and *XL*. The bladder can withstand higher stresses without compensating for elongation, which is essential to retain urine pressure at higher volumes. We believe that the high variability in the data, as indicated by Table 1, is due to the limited number of EC frogs and inherent biological- and age-related variations.

TEM and histological staining illustrated the relationship between microarchitecture and the mechanical characteristics of the tissues. The typical crimp pattern observed in H&E and trichrome stains were also found in other elastic tissues [9]. Evident in Figure 3 is the lack of large muscle bundles in *EC* tissues. Therefore, the large muscle bundles likely contribute to the tissues’ higher UTS. Furthermore, the more crimped structure found in the *EC* suggests a greater elongation when stretched. The nearly linear collagen structure found in *Xenopus* tissues (Figure 3 and Figure 4) has clear linear collagen bundles, which restrict the tissues’ ability to elongate. In combination with the stress vs. strain behaviors, the crimped collagen structure is critical to the extensibility of the tissue. However, collagen fibers from the skin of the leg were less compact, which explains the lower stress values and lower strain at failure.

Further investigations with high magnification TEM show a sophisticated collagen microstructure in the male *EC* gular tissue. Firstly, it displayed increased folding of the crimp structure, with two folds of crimp angles of 80° and 70°, compared to the female, with a single fold of crimp angle of 77°. The compact collagen allows the tissue to stretch with less force and to a higher degree. Secondly, the collagen bundles can be observed to alternate between a circular and longitudinal shape, suggesting an out-of-plane zig-zag structure. This structure likely provides the tissue with greater extensibility because the collagen bundles have additional degrees of freedom to realign in space.

Additionally, each collagen strand twists about its central axis, allowing it to unwind during elongation for higher extension (Figure 5). Lastly, the layering structure throughout the cross-section of the tissue has an alternating round and longitudinal shape, indicating a mesh-like structure. This technique is documented to aid in dissipated energy during loading [23]. Taken altogether, the gular tissue of the male *EC* has a compact three-dimensional structure that allows collagen bundles to extend significantly. Its female counterpart also displayed these features but to a lesser extent. The crimp structure was less compact, indicated by the larger curvature seen in Figure 5B. The unique features found in *EC* were not present in the *Xenopus* frogs but, instead, many locations were identified to be potential areas of stress concentration and elongation inhibition. Figure 5C,D show apparent discontinuities in the overall collagen structure in the *Xenopus* gular tissues. The discontinuities show clear breaks where the collagen aligned parallel to the tissue is impeded by collagen aligned perpendicularly. These areas are a likely cause of the higher secant modulus as they are the site of stress concentration during loading. Architecturally, *EC* tissue is unique because of its continuous hierarchical collagen structure. We believe that the multi-dimensional crimp structure allows for easy elongation of the tissue in all directions, which is key to mate calling. The difference between male and female gular tissue lies in the compactness of the structure, where a compact crimp allows greater elongation under lower forces. This property is also known as the “crimp angle” and is often used to describe the collagen structure in human tendons [24,25,26]. As mentioned in previous studies, the crimped collagen structure was determined to act as a recoiling system during muscle relaxation. This structure in the gular tissue likely serves a similar purpose in recoiling the tissue post-inflation and working in tandem with elastin.

The lamina propria in the rat bladder consisted of collagen interspersed between muscle bundles (Figure 3 and Figure 4). This feature is in clear contrast to the frog tissues, where *EC* tissues consisted of no muscle, and *XM*/*XL* had muscle bundles set apart from a collagen layer. Both TEM and histology showed that collagen in the rat bladder was less compact and less regularly structured compared to frog tissues.

As expected, the biochemical analysis showed a higher concentration of elastin in the *EC* tissues than in *Xenopus* tissues (see Figure 7). A high elastin content is one of the major contributing factors to elastic tissues and plays a key role in elongation. Male and female *EC* gular tissue had 58 ± 17 and 64 ± 10 μg/mg of wet tissue, respectively, whereas *XL* and *XM* only had 22 ± 5 and 42 ± 12 μg/mg of wet tissue, respectively. The higher concentration of elastin present in *EC* gular tissue sets it apart, bio-compositionally, from *XL* and *XM*. The biocomposition translates to a higher elongation, evident in the mechanical characteristics shown in Figure 2. Furthermore, male and female *EC* gular tissues have similar elastin concentrations, but the male *EC* has larger elongation. This emphasizes the benefits of the collagen ultrastructure for the mechanics of the tissue.

Although very dissimilar under histology stains, TEM was able to uncover similar collagen ultrastructure with the rat bladder. As shown in Figure 6A,B, the rat bladder and *EC* gular skin share ultrastructural features such as out-of-plane zig-zag and crimp structures. Additionally, *EC* gular skin and rat bladder had similar average elongations, of 398% and 412%, respectively. This clearly shows the importance of the collagen ultrastructure on the elongation of the tissue. Furthermore, rat bladder contained 485 ± 122 μg elastin/mg of wet tissue, eight-fold higher than *EC* gular skin. The significantly higher amount of elastin in the rat bladder did not translate to higher elongation. However, an abundance of elastin quickly recoils collagen bundles during contraction. [13] These results show that this unique collagen ultrastructure or high amounts of elastin can lead to higher elongation of a material. Further investigations are required in order to elucidate the individual impact of high elastin content and various collagen ultrastructures (i.e., crimp or helical) on compliance.

Tensile tests on these tissues show the much higher UTS of the gular tissue compared to that of the leg. This is counter-intuitive because of the gular tissue’s lower collagen content; however, the increase in mechanical strength can be attributed to the hierarchical microstructure seen in the male *EC*. Shown in a previous study [27], the microstructure of the material has a significant role in the mechanical behavior of the material. Evidently shown here is that reducing performance compromises is one of the key reasons behind intricate microarchitectures. The layered structure of the collagen provides the tissue with much of its strength [27,28,29,30], while the waviness enhances tissue elongation. This hierarchical structure provides a balance between tensile strength and compliance, two mechanical properties that are not often positively correlated. As shown in a previous study [13], elastin is only regionally present in the bladder, and the helical shape of collagen provides much of the bladder’s compliance. While collagen type I provides the bladder with its strength, collagen type III and elastin provide its compliance [13,31,32,33,34,35]. The unique conformation of collagen type III and elastin provide recoverable deformation due to hydrophobic interactions. This conformation may not be required to achieve the same biomechanical action, as gular skin did not contain coiled collagen or a high elastin content. The compliance of male *EC* tissue is a result of its tissue-specific collagen ultrastructure, thus reinforcing the importance of material architecture in bulk material properties. The functional similarities between the gular tissue and the bladder prove that the same fundamental mechanisms can be used in biomaterial design for bladder reconstruction.

Taken altogether, we believe that the collagen architecture shown here in EC gular skin tissue can be relevant in tissue engineering of large-deforming compliant tissues. The unique orthogonal arrangement of collagen layers can also be potentially considered for corneal tissue engineering [36]. Future work will involve recapitulating this structure using 3D bioprinting techniques to develop reinforced hydrogels [37,38,39]. Additionally, the area of decellularized tissues in tissue engineering has generated considerable attention in recent works [40,41]. Decellularized EC gular tissues have the potential to act as a temporary functional graft, although additional experiments will need to be conducted to validate its potential.

## 4. Materials and Methods

### 4.1. Specimen Collection

Euthanized male and female *E. Coqui* were obtained from Atlanta Botanical Garden (Atlanta, GA, USA). The females were tested as a species-specific control as females do not tend to inflate their gular tissue. The number of biological replicates was limited because these frogs were taken from live populations, and excessive sampling of frogs causes ecological instability. In total, four male ECs and two female ECs were collected for all the experiments. Although the age of the frogs may influence the mechanics of the tissue, *E. Coqui* was obtained from a population, and an exact age could not be determined. The estimated age was between 1 and 5 years. In total, four euthanized male *X. Laevis* and four male *X. Muelleri* were obtained from Xenopus Express Inc. These species were used as negative controls as they do not display any external tissue inflation during calls. All specimens were euthanized on-site before either fixation or freezing and were transported overnight. Samples used for tensile testing were received frozen in ice and immediately dissected for sample collection. Samples were then stored at −20 °C until testing was performed. Fixed samples were used for histology and microscopy, while frozen samples were used for tensile tests and biochemical assays. The skin tissue of the desired regions was dissected in the laboratory with surgical scissors and scalpels. The specimen’s gular and leg skin tissue were of particular interest; areas examined can be seen in Figure 1b. Rat bladders were dissected in accordance with protocols approved by the Johns Hopkins University Animal Care and Use Committee (RA17M330, 7/23/2018).

### 4.2. Uniaxial Tensile Test

We determined the mechanical properties of the unfixed and hydrated skin tissues by conducting uniaxial tensile tests using the Instron Tensile testing equipment (MTS Criterion™ 40). Samples (*n* = 3) per group (male *EC:* three biological repeats; female *EC:* two biological repeats; male *XL:* three biological repeats; male *XM:* three biological repeats) for both gular skin and leg were cut into small rectangular pieces and measured for their thickness, width, and length. Cut samples ranged from 1 mm to 3 mm wide and 4 mm long. Average sample thickness (0.1–0.4 mm) was measured using an electronic caliper, which varied due to differences in species and the site of extraction (gular vs. leg). Generally, *EC* samples were thinner than those of *XL*/*XM*. The sample dimensions were limited to the size of the frogs, as EC is extremely small (~1.5 inches for male). Reference state dimensions were established after mounting the tissue onto the apparatus and tared before testing began. Preconditioning was not performed prior to tensile loading. We also assumed that the tissues would behave isotropically under tensile loading. We acknowledge that the biaxial test would be more predictive of tissue expansion; however, because of the small size of *EC*, we opted for comparative uniaxial analysis. Each side of the cut samples was then glued in between two pieces of thick paper to increase the surface area that is clamped onto the instrument, similar to a study conducted by Dahms et al. [42]. The strain was calculated using the distance between the clamps. The samples were extended with a strain rate of 0.5 mm/min, using a 5N load cell. Engineering stress, membrane tension, strain, and secant modulus (at 20% strain) were calculated and visualized with GraphPad Prism 8.0. Secant moduli were calculated using the stress and strain values before and after 20% strain.

### 4.3. Histology

Tissue samples were washed in Phosphate Buffer Saline (PBS), formalin-fixed, and paraffin-embedded for staining using hematoxylin and eosin (H&E) and Masson’s trichrome, in accordance with our previous study [43]. Paraffin blocks were sectioned at 6 microns onto glass slides for brightfield microscopy imaging.

### 4.4. Transmission Electron Microscopy

Samples were fixed in 3% paraformaldehyde, 1.5% glutaraldehyde, 5 mM CaCl_2_, 2.5% sucrose, and 0.1% tannic acid in 0.1 M sodium cacodylate buffer, pH 7.2. After buffer rinse, samples were fixed in 1.0% osmium tetroxide for 1 h on ice in the dark. Following a rinse with distilled water, the samples were stained with 2.0% aqueous uranyl acetate (0.22 µm filtered) for 1 h in the dark, dehydrated using a graded series of ethanol, and embedded in Eponate 12 resin (Ted Pella, Redding, CA, USA). Samples were polymerized for 2–3 days at 37 °C and were stored at 60 °C overnight. Thin sections, 60–90 nm, were cut at a depth of 50 µm from the surface with a diamond knife using a Reichert-Jung Ultracut E ultramicrotome and placed on naked copper grids and were stained with 2% uranyl acetate in 50% methanol and observed using a Philips/FEI BioTwin CM120 TEM.

### 4.5. Crimp Angle Measurement 

The crimp angle analyzed through TEM imaging of unloaded samples was calculated by drawing a line along the straight edges of a collagen layer and measuring the angle between the two lines using ImageJ software (NIH). Measurement lines are shown in green in Figure 5E,F for reference.

### 4.6. Collagen and Elastin Content

The collagen and elastin concentrations in both gular and leg tissues (*n =* 3) were determined using Biocolor Sircol™ Insoluble Collagen and Biocolor Fastin™ Elastin assays (Accurate Chemical, Westbury, NY). Briefly, samples ranging from 0.1 to 4.0 mg wet weight were thawed and dissociated in the fragmentation reagent provided by the kit. We performed tissue fragmentation for 3 h at 65 °C under intermittent vortex mixing. Dissociated collagen was collected through centrifugation and dyed using the Sircol Dye Reagent for 30 min, followed by centrifugation. Excess liquid was drained and exposed to an acid-salt wash to remove the unbound dye and centrifuged to collect the collagen-dye pellet and remove excess dye. The alkali reagent was added and vortexed to remove the bound dye and immediately used for colorimetric absorbance measurements. Elastin was extracted according to the protocol described briefly here. Elastin was extracted using 0.25 M oxalic acid at 100 °C for 2 h and precipitated using the provided precipitating agent. The solution was centrifuged to form an elastin pellet, exposed to the dye reagent for 90 min, and vortexed intermittently. Following centrifugation, the elastin-bound dye was released using the provided dissociation reagent and used immediately for colorimetric absorbance reading. Results from both assays were expressed in micrograms of collagen or elastin per milligram of the wet tissue sample.

### 4.7. Statistical Methods

Statistical analysis was conducted on GraphPad Prism 8.0 using two-way ANOVA (Holm-Sidak) for biochemical assays and two-way ANOVA (Dunnett’s) for tensile properties, with an α value of 0.05. All sample groups were tested against each other for biochemical assays, and tensile properties were compared against the rat bladder as a control.

## 5. Conclusions

In conclusion, our results emphasize the role of protein microarchitecture on the mechanical properties of a material. We first illustrated the unique mechanical properties of the male *EC* gular tissue, contrasted with other tissue types, other species of frogs, gender, and rat bladder. The high elongation and moderate UTS demonstrated the tissues’ ability to reduce performance trade-offs. That is, the tissue was able to elongate while maintaining higher stresses. Importantly, the specific multi-dimensional hierarchical collagen structure allowed the tissue to elongate as well as maintain moderately higher stresses. Together, the two components allow the unique functionality of the gular tissue to inflate and withstand the internal air pressure during calling. The elongation similarities of *EC* gular skin and rat bladder are reinforced by the ultrastructural likeness of the two tissues. Taken together, these findings demonstrate the potential of the *EC* gular skin as a novel biomimetic example for developing materials for tissue engineering of large deforming tissues, such as the bladder. The characterization of this microarchitecture can provide a simple template for future artificial biomaterial scaffold designs for regenerative medicine applications such as biomimicry in 3D printed bladder tissues.

## Figures and Tables

**Table 1:** Summary of the mechanical properties.

Specimen	Tissue	UTS (kPa)	Strain at Peak Stress (%)	Breaking Strain (%)	Secant Modulus (20% strain) (kPa)
		Mean ± SD (*p*)	Mean ± SD (*p*)	Mean ± SD (*p*)	Mean ± SD (*p*)
Coqui (M)	Gular	1263 ± 134 (0.188)	187 ± 22 (0.999)	398 ± 86 (0.999)	373 ± 194 (0.808)
	Leg	193 ± 40 (0.006)	83 ± 53 (0.999)	348 ± 88 (0.999)	318 ± 333 (0.762)
Coqui (F)	Gular	2142 ± 1789 (0.861)	226 ± 43 (0.999)	337 ± 83 (0.999)	786 ± 1052 (0.991)
	Leg	936 ± 1265 (0.078)	101 ± 59 (0.999)	252 ± 39 (0.999)	1475 ± 1823 (0.999)
XL	Gular	4461 ± 2215 (0.330)	78 ± 12.5 (0.999)	104 ± 17(0.999)	3117 ± 1400 (0.143)
	Leg	2553 ± 1775 (0.996)	61 ± 23 (0.999)	108 ± 12 (0.999)	4863 ± 2118 (0.003)
XM	Gular	4156 ± 1973 (0.581)	271 ± 70 (0.999)	350 ± 9 (0.999)	807 ± 668 (0.993)
	Leg	2524 ± 560 (0.932)	126 ± 16 (0.999)	175 ± 28 (0.999)	2877 ± 2265 (0.258)
Rat	Bladder	2985 ± 853	233 ± 28	412 ± 163	1288 ± 850

**Table 2:** Summary of elastin and collagen content in tissue samples.

		Elastin (μg/mg Wet Tissue)	Collagen (μg/mg Wet Tissue)	Elastin/Collagen Ratio
Specimen	Tissue	Mean ± SD	Mean ± SD	
Female Coqui	Gular	64.5 ± 9.8	234.5 ± 86.7	0.27
	Leg	39.0 ± 9.8	183.1 ± 45.9	0.21
Male Coqui	Gular	58.5 ± 16.9	202.0 ± 41.3	0.29
	Leg	48.8 ± 14.8	325.0 ± 69.5	0.15
XL	Gular	22.1 ± 5.2	377.8 ± 105.3	0.06
	Leg	26.0 ± 4.6	396.7 ± 36.3	0.07
XM	Gular	42.1 ± 11.9	400.8 ± 36.5	0.10
	Leg	37.9 ± 1.4	262.1 ± 24.3	0.14
Rat	Bladder	484.8 ± 121.9	96.1 ± 19.4	5.05

## Abbreviations

UTS	Ultimate Tensile Strength
EC	Eleutherodactylus Coqui
XL	Xenopus Laevis (XL)
XM	Xenopus Muelleri
TEM	Transmission Electron Microscopy
SEM	Scanning Electron Microscopy
H&E	Haemotoxylin and Eosin
